# An open‐label randomized study of the relative absorption of gastro‐resistant risedronate taken fasted or with food versus immediate‐release risedronate

**DOI:** 10.1002/prp2.957

**Published:** 2022-05-08

**Authors:** Diane Kleinermans, Andrew Joyson, Heather Wray

**Affiliations:** ^1^ Formerly Covance Clinical Research Unit Ltd Leeds UK; ^2^ Formerly Pharmaceutical Profiles Ltd Ruddington UK

**Keywords:** bisphosphonate, osteoporosis, pharmacokinetics, risedronate

## Abstract

Patients with osteoporosis often take oral bisphosphonates with food, rendering these medications ineffective. This study compared the relative absorption of four formulations of gastro‐resistant (GR; formulations 1–4) risedronate 35 mg versus immediate‐release (IR) risedronate 35 mg taken fasted. Secondarily, it compared the relative absorption of GR formulations administered fed and fasted, and determined the site of disintegration. Healthy participants (*N* = 160) were randomized to one of nine treatment groups: IR risedronate taken fasted (group A) or formulations 1–4 taken fasted or fed (groups B–I). Fasted groups fasted for 8 h pre‐dose and 4 h post‐dose. Fed groups fasted for 7.5 h, then took risedronate with breakfast. Urine was collected until 72 h post‐dose and analyzed using liquid chromatography. From each group, up to seven participants underwent scintigraphic monitoring to assess tablet disintegration. The percentage of total dose recovered in urine (A’_e_) was ~0.5% for group A. The A’_e_ of formulations 1–4 taken fasted was 0.220% (90% confidence interval 0.124–0.389), 0.298% (0.122–0.730), 0.154% (0.090–0.264), and 0.108% (0.051–0.231), respectively. With food, the A’_e_ of formulation 1 decreased least versus fasted (−27%) compared with the A’_e_ of formulations 2, 3, and 4 (−73%, −80%, and −65%, respectively). Formulations 1–3 disintegrated in the small intestine, formulation 4 closer to the large intestine. All GR formulations were well tolerated and in line with the known safety profile for IR risedronate. Formulation 2 had the highest absorption when taken fasted, whereas the absorption of formulation 1 was least affected by food.

## Graphical abstract

In clinical practice, at least 30% of patients find it difficult to comply with the fasting instructions for oral bisphosphonates. This study compared the relative absorption of four formulations of gastro‐resistant risedronate 35 mg (formulations 1–4) taken fasted or with food vs. immediate‐release risedronate 35 mg taken fasted.

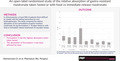

## 1 INTRODUCTION

Osteoporosis is an age‐related skeletal disease common in post‐menopausal women and is characterized by decreasing bone mass and increased risk of fractures.^1^, ^2^ It affects approximately 20 million people in six of the largest countries in Europe, the majority of whom are female.^2^ Oral bisphosphonates (BPs), such as risedronate, alendronate and ibandronate, are routinely recommended as first‐line treatments for patients with osteoporosis.^1^, ^3^, ^4^ In clinical trials, risedronate has demonstrated significant efficacy in reducing vertebral and non‐vertebral fractures compared with placebo,^5^, ^6^ a finding that was further confirmed through real‐world evidence.^7^, ^8^

When taken according to the dosage instructions, the absolute bioavailability of risedronate is <1% with oral administration.^9^ If taken with food, the bioavailability of risedronate and other oral BPs is thought to be close to zero.^10^, ^11^, ^12^ This may be because they form insoluble chelates with cations in food, such as calcium and magnesium.^10^ Therefore, risedronate and other oral BPs must be taken on an empty stomach after an overnight fast with a ≥30‐min wait before any food or drink.^13^, ^14^, ^15^

In clinical practice, at least 30% of patients find it difficult to comply with the fasting instructions for oral BPs.^11^, ^16^ Recurrent non‐compliance with fasting instructions means that patients may be more vulnerable to osteoporotic fractures.^17^ A gastro‐resistant (GR) formulation of risedronate that can be taken with food may overcome the fasting challenges that patients face when taking oral BPs.

This study investigated the relative absorption of four modified‐release formulations of risedronate GR 35 mg under fed and fasted conditions versus the immediate‐release (IR) formulation of risedronate 35 mg under fasted conditions. The site of tablet disintegration was also assessed in a subset of participants.

## 2 MATERIALS AND METHODS

### 2.1 Study population

Healthy male and female participants were included if aged 40–70 years with a body mass index <32 kg m^–2^. Female participants must have been non‐lactating, and surgically sterile or postmenopausal (12 consecutive months without menses). All patients had to provide written informed consent. Participants were excluded if they had (1) any disease or surgery known to alter gastrointestinal (GI) structure or function; (2) a history of GI disease or GI surgery (apart from appendectomy and hernia repair that did not require bowel resection); (3) acute diarrhea or constipation (within 14 days prior to the predicted first study day); (4) a history of cancer within the past 5 years (apart from basal cell carcinoma with a 6‐month remission or cervical carcinoma with a 12‐month remission); (5) creatinine clearance (CrCl) of <60 ml min^–1^; (6) a history of substance abuse, a positive urine screen for drugs, or elevated alcohol consumption or tobacco use; (7) a known allergy to BPs or had used BPs within 6 months prior to dosing; (8) used any excluded prescription drug or herbal remedy within 14 days before dosing, or used any excluded medications that altered GI pH or motility within 7 days before dosing (aminoglutethimide, amiodarone, antipyrine, atorvastatin, barbiturates, carbamazepine, chloral hydrate, cimetidine, clarithromycin, desipramine, dexamethasone, diltiazem, diphenhydramine, erythromycin, ethosuximide, fluconazole, fluoxetine, fluvoxamine, griseofulvin, imipramine, itraconazole, ketoconazole, lansoprazole, metoclopramide, mibefradil, midazolam, nefazodone, nifedipine, omeprazole, paroxetine, phenobarbital, phenylbutazone, phenytoin, pioglitazone, progesterone, rifabutin, rifampin, sertraline, St. John's wort, testosterone, troleandomycin, verapamil, zafirlukast); (9) a positive serum pregnancy test; (10) a positive screen for hepatitis B/C or human immunodeficiency virus.

During the study, participants were removed if they withdrew voluntarily, if it was in the best interest of the participant (as determined by the investigator), if an illness developed that interfered with evaluation of the study drug, if any excluded concomitant medication was used, or if the participant did not comply with urine collection requirements.

This study was conducted in line with the ethical principles outlined in the Declaration of Helsinki. Ethics approval was obtained from the Huntingdon Local Research Ethics Committee, Cambridge, UK. All participants provided written informed consent prior to the study.

### 2.2 Study design

This was a randomized, open‐label, single‐dose, two‐centre, parallel‐group study. Participants were screened within 28 days before admission to the study site and assigned to one of nine treatment groups in equal numbers using block randomization (block size: 9; Table 1). Sample size was determined based on unpublished data^18^ indicating that the percentage of the dose recovered in urine (A’_e_) for the reference formulation was 0.40% with a percent coefficient of variation (CV%) of 100%. Assuming the CV% of the test formulations are also 100%, 15 subjects per treatment group provides 80% power for a one‐sided *t*‐test (*α* = 0.10) to conclude that the A’_e_ % of a test formulation is ≥45% of that of the reference formulation.

**TABLE 1:** Summary of treatment groups, including study drug received and state of administration (fasted or fed)

	Core type	Active ingredient	pH trigger	Enteric coating level	Administration	Treatment group
	Immediate release^a^	35 mg risedronate	None	None	Fasted	A
Formulation 1	Gastro‐resistant, delayed release	35 mg risedronate/100 mg edetate sodium	5.5	Low coating^b^	Fasted	B
					Fed	C
Formulation 2	Gastro‐resistant, delayed release	35 mg risedronate/100 mg edetate sodium	5.5	High coating^c^	Fasted	D
					Fed	E
Formulation 3	Gastro‐resistant, sustained release	35 mg risedronate/100 mg edetate sodium	5.5	High coating^b^	Fasted	F
					Fed	G
Formulation 4	Gastro‐resistant, delayed release	35 mg risedronate/100 mg edetate sodium	7.0	High coating^d^	Fasted	H
					Fed	I

^a^ Currently marketed immediate‐release tablet.

^b^ 10% methacrylic acid copolymer type C coating.

^c^ 30% methacrylic acid copolymer type C coating.

^d^ Methacrylic acid copolymer type B coating.

The study consisted of a single 72 h period: participants were admitted to one of two clinical trial units (located in Leeds and Nottingham, UK) on the evening of day 0 (prior to dosing) and were observed until 36 h after dosing (day 2). Participants returned to the clinic 48 h (day 3) and 72 h (day 4) after study drug administration.

For fasted administration, participants fasted from midnight until dosing at ~8:00 am and continued to fast until 4 h post‐dose. For fed administration, participants fasted from midnight until ~7:30 am, when they ate a high‐fat breakfast (two slices of white toast, two pats of butter, two eggs fried in butter, two slices of bacon, 113 g of hash brown potatoes and 226 g of whole milk). Participants were dosed at ~8:00 am following this meal. The risedronate tablet was swallowed with 240 ml of plain water; water was restricted for 1 h before and after dosing.

This was an exploratory study. The primary objective was to compare the relative absorption of GR versus IR risedronate under fasted conditions. The secondary objectives were to compare the relative absorption of GR formulations when administered under fed and fasted conditions, as well as to determine the site of disintegration of GR tablets.

### 2.3 Pharmacokinetic and bioanalytical methods

Urine samples were collected at baseline and pooled post‐dose (0–24 h [24 h time point], 24–48 h [48 h], 48–72 h [72 h]). Urine samples were collected in polypropylene containers and refrigerated at 4°C between collections until the end of the collection period. At the end of the collection period, urine was mixed thoroughly and transferred to two polypropylene tubes in 10 ml aliquots. These samples were frozen at −20°C until analysis. Contact of urine with unsiliconized glass or metal surfaces was avoided to prevent adsorption of the drug.

SFBC Anapharm determined the cumulative amount of risedronate recovered (A_e_, mg) and the A’_e_ (%) in pooled urine samples using validated liquid chromatography with tandem mass spectrometry (LC/MS/MS; Anapharm Method SOP ANI 8809.01). The analyte risedronic acid and its corresponding internal standard, risedronic acid−d_4,_ were extracted from human urine by solid phase extraction and derivatization procedures. The sample was subjected to a normal‐phase, high‐performance liquid chromatographic analysis on a Chirobiotic™ T 50 × 4.6 mm column. The analyte and internal standard were detected and quantitated by tandem mass spectrometry operating under multiple reaction monitoring LC/MS/MS conditions. Quantitation was done by the ratio of peak area of the analyte to the internal standard and comparing that value to those on a standard curve. Standards and quality control samples were quantified as risedronic acid and study samples were reported as risedronate sodium. A molecular weight correction factor of 1.078 was applied to all study specimen results to convert the concentration obtained in risedronic acid to nanograms of risedronate sodium per ml of urine. The concentrations of the analyte in the specimens were determined from a weighted linear 1/*x* ^2^ regression of the calibration curve of spiked matrix standards. The range of quantitation was 0.2–200 ng risedronic acid/ml (nominal) using a 200 μl urine sample. The actual lower limit of quantification of risedronic acid/ml of urine during study sample specimen analysis was 0.20 ng/ml of urine (0.216 ng/ml when corrected to risedronate sodium).

### 2.4 Scintigraphic monitoring

A subset of up to seven participants per treatment group (males and surgically sterile females only) underwent scintigraphic monitoring to assess gastric emptying and tablet disintegration. Participants were excluded if they had participated in a study using radionuclides within the previous 3 months. The parameters assessed were: (1) gastric emptying time, (2) time and site of disintegration onset, (3) time and site of complete disintegration.

GR tablets were labeled with ~1 MBq of samarium oxide (^152^Sm) and subjected to neutron activation (^152^Sm → ^153^Sm). The position of the tablet in the GI tract was determined in relation to an anterior external marker (0.1 MBq of ^99m^technetium) positioned in the same transverse plane as the pylorus. Anterior scintigraphic images (~50‐s duration) were recorded using a gamma camera (General Electric Maxicamera or ADAC Forte) with a 40‐cm field of view. Images were recorded until tablet disintegration (every 15 min for 12 h post‐dose, then every 30 min up to 16 h post‐dose and every hour until 24 h post‐dose). Participants could freely walk around the clinical unit.

### 2.5 Safety assessment

Safety was assessed through physical examination, vital signs, and adverse events (AEs) monitoring by the participants, the investigator or study site personnel. Suspected AEs were evaluated and appropriate treatment and follow‐up provided as necessary. Participants with clinically significant AEs remained under medical supervision until the AE resolved, stabilized or was no longer serious enough to warrant follow‐up (as assessed by the investigators).

### 2.6 Statistical analysis

All statistical analyses were conducted using SAS^®^ Version 8 in the HP‐UNIX environment or Microsoft^®^ Excel (Office 365 version 16.54). Continuous data (such as A_e_) were summarized using descriptive statistics (e.g., mean, SD, median, minimum, maximum). Categorical data (such as AEs) were summarized by treatment condition in counts and percentages. Nominal 90% confidence intervals (CIs) were constructed for ratios of pharmacokinetic parameters of the fasted arm of each test formulation (groups B, D, F, and H) to the IR formulation (treatment group A). Similar analyses were performed for the fed arm of each test formulation (groups C, E, G, and I) versus its respective fasted arm (groups B, D, F, and H). Data were examined for site effects and analyses were conducted accordingly.

### 2.7 Nomenclature of targets and ligands

Key protein targets and ligands in this article are hyperlinked to corresponding entries in http://www.guidetopharmacology.org, the common portal for data from the IUPHAR/BPS Guide to PHARMACOLOGY (Harding et al., 2018),^19^ and are permanently archived in the Concise Guide to PHARMACOLOGY 2019/20 (Alexander et al., 2019).^20^

## 3 RESULTS

### 3.1 Patient characteristics

**FIGURE 1:**
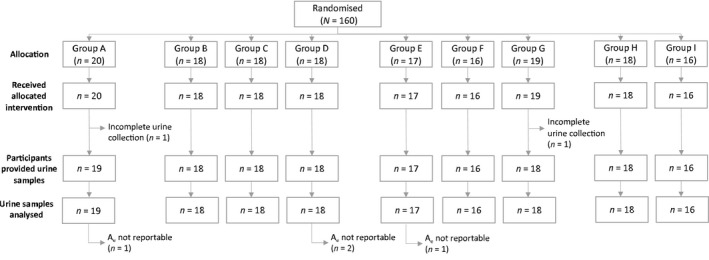
Disposition of study participants. A_e_, amount of risedronate recovered in urine (mg).

In total, 160 participants were randomized. Two subjects were withdrawn due to incomplete urine collection and 158 participants completed the study (Figure 1). Baseline characteristics were similar across treatment groups (Table 2). Participants had a median age of 49.5 years with a median CrCl of 81.3 ml min^–1^. The majority of participants were male and Caucasian.

**TABLE 2:** Participant demographics

Group	A (*n* = 20)	Formulation 1		Formulation 2		Formulation 3		Formulation 4		Total (*N* = 160)
		B (*n* = 18)	C (*n* = 18)	D (*n* = 18)	E (*n* = 17)	F (*n* = 16)	G (*n* = 19)	H (*n* = 18)	I (*n* = 16)	
Age (years), median (range)	47 (40–70)	50 (40–69)	47.5 (40–70)	51.5 (40–69)	53 (40–60)	50.5 (41–68)	52 (40–61)	45 (40–62)	44 (40–69)	49.5 (40–70)
Male, *n* (%)	16 (80.0)	15 (83.3)	13 (72.2)	13 (72.2)	13 (76.5)	12 (75)	13 (68.4)	15 (83.3)	10 (62.5)	120 (75)
Race, *n* (%)										
Caucasian	19 (95)	18 (100)	17 (94.4)	18 (100)	17 (100)	16 (100)	17 (89.5)	18 (100)	14 (87.5)	154 (96.25)
Black	1 (5)	0	1 (5.6)	0	0	0	1 (5.25)	0	1 (6.25)	4 (2.5)
Asian	0	0	0	0	0	0	0	0	1 (6.25)	1 (0.625)
Mixed‐race	0	0	0	0	0	0	1 (5.25)	0	0	1 (0.625)
Height (cm), median (range)	173 (161–182)	172.5 (160– 186)	172 (160–194)	172.5 (152–183)	170 (158–187)	172.5 (161–183.1)	171 (152–180)	173 (155–187)	167.5 (150–193)	172 (150–194)
Weight (kg), median (range)	76.4 (64.9–93.9)	78.95 (62.3–97.8)	74.55 (59.2–93.6)	78.0 (54.6–99.6)	76 (65.9–97.6)	77.7 (61.0–98.1)	71.4 (53.7–97.2)	79.4 (57.0–95.7)	72.1 (49.6–95.4)	76.4 (49.6–99.6)
CrCl (ml min^–1^), median (range)	85.5 (54.6–106.9)	79.5 (61.7–116.4)	78.2 (61.2–109.5)	78.85 (55.7–101.5)	86.1 (57.8–101.5)	83.95 (59.9–112.5)	81.3 (56.3–113.8)	87.9 (59.2–108.5)	78.55 (60.7–118.6)	81.3 (54.6–118.6)

Abbreviation: CrCl, creatinine clearance.

### 3.2 Pharmacokinetic results

**FIGURE 2:**
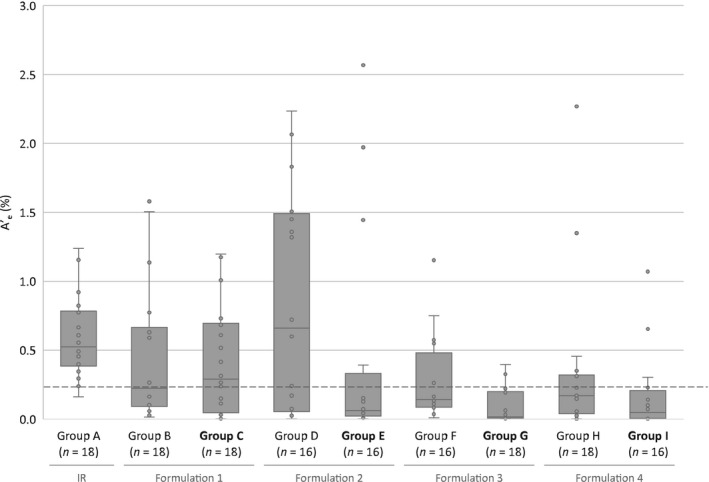
A’_e_ up to 72 h after dosing with different formulations of risedronate 35 mg. Groups highlighted in bold received risedronate after food, whereas all other groups received it in a fasted state. Dashed line represents a 50% reduction of the group A median A’_e_. The top and bottom lines of each box represent the upper quartile and lower quartile, respectively, and the mid‐line represents the median value. The whiskers show the minimum and maximum values. Circles represent A’_e_ value for individual participants. A’_e_, percentage of total dose of risedronate given recovered in urine; IR, immediate release.

The A_e_ (in mg) was assessed over a 72‐h period after dosing and expressed as A’_e_ (%) (Table 3 and Figure 2). This showed that ~0.5% of the dose was recovered in participants who received IR risedronate 35 mg.

**TABLE 3:** Summary of A’_e_ in all treatment groups assessed over 72 h

Group	A (*n* = 18)^b^	Formulation 1		Formulation 2		Formulation 3		Formulation 4	
		B (*n* = 18)	C (*n* = 18)	D (*n* = 16)^b^	E (*n* = 16)^b^	F (*n* = 16)^c^	G (*n* = 18)	H (*n* = 18)	I (*n* = 16)
A’_e_ (%), geometric mean (90% CI)	0.520 (0.418–0.646)	0.220 (0.124–0.389)	0.161 (0.072–0.356)	0.298 (0.122–0.730)	0.082 (0.034–0.196)	0.154 (0.090–0.264)	0.032 (0.016–0.062)	0.108 (0.051–0.231)	0.038 (0.016–0.090)
A’_e_ ratio vs. group A (90% CI)		0.423 (0.233–0.766)	0.309 (0.138–0.689)	0.573 (0.246–1.335)	0.158 (0.069–0.359)	0.296 (0.173–0.508)	0.061 (0.031–0.121)	0.208 (0.097–0.448)	0.072 (0.032–0.165)
A’_e_ ratio fed vs. fasted (90% CI)^a^			0.731 (0.282–1.893)		0.275 (0.082–0.921)		0.20 (0.088–0.479)		0.346 (0.114–1.059)
A’_e_ (%), median	0.523	0.224	0.290	0.661	0.062	0.141	0.017	0.169	0.049
A’_e_ (%), minimum	0.163	0.016	0.002	0.002	0.002	0.009	0.002	0.002	0.002
A’_e_ (%), maximum	1.239	1.579	1.197	2.235	2.568	1.153	0.396	2.269	1.070
A’_e_ (%), IQR	0.400	0.575	0.648	1.438	0.310	0.395	0.193	0.282	0.202

Abbreviations: Cl, confidence interval; IQR, interquartile range.

^a^ The ratio of fed to fasted for each modified‐release formulation is based on the ratio of each formulation versus the immediate‐release formulation.

^b^ A’_e_ values were not calculable for some participants (group A: *n* = 1; group D: *n* = 2; group E: *n* = 1).

^c^ A’_e_ values for one participant in group F were not reportable at 72 h—values were carried forward from 24–48 h interval. A’_e_, percentage of total dose of risedronate given recovered in urine.

Compared with risedronate IR, the A’_e_ was lower for all participants who received GR formulations. The A’_e_ of formulation 1–4 was 0.220% (90% CI 0.124–0.389), 0.298% (90% CI 0.122–0.730), 0.154% (90% CI 0.090–0.264), and 0.108% (90% CI 0.051–0.231), respectively.

When comparing the A’_e_ of GR formulations administered under fasted and fed conditions, formulation 1 was least affected by food (group B vs. C). This manifested as a ~27% decrease in A’_e_ versus the fasted condition (ratio 0.731 [90% CI 0.282–1.893]), whereas other GR formulations showed a 73% (formulation 2; group D vs. E ratio 0.275 [90% CI 0.082–0.921]), 80% (formulation 3; group F vs. G ratio 0.20 [90% CI 0.088–0.479]) and 65% (formulation 4; group H vs. I ratio 0.356 [90% CI 0.114–1.059]) decrease.

### 3.3 Scintigraphic results

Gastric emptying time, onset of disintegration and complete disintegration of GR tablets in the GI tract were assessed using scintigraphic monitoring (Table 4). The mean gastric emptying time, time to initial disintegration and time to complete disintegration were all faster in the fasted state than in the fed state.

**TABLE 4:** Mean times and locations of onset and complete disintegration of different formulations of GR risedronate

Formulation	Group/administration	Time to gastric emptying (h) Mean (±SD)	Time to onset of disintegration (h) Mean (±SD)	Time to complete disintegration (h) Mean (±SD)	Onset of disintegration location	Complete disintegration location
Formulation 1	Group B: Fasted (*n* = 7)	0.53 (0.43)	2.04 (0.38)	3.77 (1.04)	Proximal/distal small bowel	Distal small bowel/ascending colon
	Group C: Fed (*n* = 7)	9.80 (7.00)	10.70 (7.59)	11.54 (8.47)	Proximal/distal small bowel	Proximal small bowel/ascending colon
Formulation 2	Group D: Fasted (*n* = 7)	0.95 (0.59)	4.03 (0.83)	4.34 (0.78)	Distal small bowel/ascending colon	Distal small bowel/ascending colon
	Group E: Fed (*n* = 6)	11.64 (7.55)	13.50 (7.89)	13.83 (8.08)	Proximal small bowel/ileocecal junction	Distal small bowel/ileocecal junction
Formulation 3	Group F: Fasted (*n* = 7)	0.65 (0.51)	2.43 (0.87)	3.16 (1.00)	Proximal/distal small bowel	Proximal small bowl/transverse colon
	Group G: Fed (*n* = 6)	10.49 (8.27)	9.95 (8.86)	10.25 (9.09)	Stomach/distal small bowel	Stomach/distal small bowel
Formulation 4	Group H: Fasted (*n* = 7)	0.74 (0.53)	6.35 (0.47)	8.00 (2.07)	Ileocecal junction/ascending colon	Ileocecal junction/ascending colon
	Group I: Fed (*n* = 5)	11.36 (6.70)	13.72 (6.74)	14.56 (7.29)	Proximal small bowel/ileocecal junction	Proximal small bowel/ascending colon

Abbreviations: GR, gastro‐resistant; SD, standard deviation.

When examining the relationship between A_e_ and site/time of disintegration, A_e_ appears to be independent of the site of release prior to the ascending colon, after which it appears to decrease. In addition, increasing time in the GI tract seemed to be associated with lower A_e_.

### 3.4 Safety

All GR formulations were well tolerated under fasted and fed conditions, with no serious AEs, discontinuation due to AE or deaths (Table 5). In total, 84 AEs were reported by 51 participants (mild severity: 65; moderate severity: 19). Of these, 49 AEs were possibly, and 4 AEs were probably, related to the study drug. There was no difference in the distribution, severity or causality of AEs according to GR formulation or whether participants received these in the fasted/fed state.

**TABLE 5:** Summary of AEs

Group, *n* (%)	A (*n* = 20)	Formulation 1		Formulation 2		Formulation 3		Formulation 4		Total (*N* = 160)
		B (*n* = 18)	C (*n* = 18)	D (*n* = 18)	E (*n* = 17)	F (*n* = 16)	G (*n* = 19)	H (*n* = 18)	I (*n* = 16)	
Participants reporting										
Any AE	5 (25)	2 (11)	6 (33)	8 (44)	3 (18)	6 (38)	8 (42)	8 (44)	5 (31)	51 (32)
Severe AE	0	0	0	0	0	0	0	0	0	0
AEs causing discontinuation	0	0	0	0	0	0	0	0	0	0
Deaths	0	0	0	0	0	0	0	0	0	0
Common AEs^a^										
Headache	1 (5)	0	0	3 (17)	2 (12)	0	1 (5)	2 (11)	1 (6)	10 (6)
Back pain	1 (5)	0	1 (6)	2 (11)	0	1 (6)	1 (5)	1 (6)	2 (13)	9 (6)
Pain in extremity	0	0	0	1 (6)	0	3 (19)	1 (5)	1 (6)	1 (6)	7 (4)
Loose stools	0	2 (11)	2 (11)	0	0	0	1 (5)	0	1 (6)	6 (4)
Myalgia	1 (5)	0	0	1 (6)	0	1 (6)	1 (5)	1 (6)	0	5 (3)
Abdominal pain	0	0	2 (11)	0	0	0	0	0	1 (6)	3 (2)
Arthralgia	0	0	0	0	0	1 (6)	1 (5)	0	1 (6)	3 (2)
Dyspepsia	0	0	1 (6)	0	2 (12)	0	0	0	0	3 (2)
Flatulence	0	1 (6)	1 (6)	0	0	0	1 (5)	0	0	3 (2)
Nausea	0	0	1 (6)	1 (6)	0	0	1 (5)	0	0	3 (2)

Abbreviation: AE, adverse event.

^a^ AEs were defined as common if they occurred in >1% of the total participant population.

## 4 DISCUSSION

Oral BPs are a mainstay of treatment for osteoporosis,^1^, ^3^, ^4^ with demonstrated effectiveness in reducing the incidence of fractures.^5^, ^6^, ^7^, ^8^ The bioavailability of oral BPs is low^9^ and is further inhibited by food.^10^, ^11^, ^12^ For this reason, patients may benefit from the availability of a GR formulation of oral BP that would allow them to take their medication with food.^21^ The aim of this study was to assess the relative absorption of four GR formulations of risedronate 35 mg under fasted conditions with IR risedronate 35 mg. In addition, this study investigated the effect of food on the relative absorption of these four GR risedronate formulations and their site of disintegration.

Participants in this study received IR risedronate 35 mg after an overnight fast and 4 h before food. Analysis of A_e_ after 72 h showed that the A’_e_ was ~0.52% of the original 35 mg dose (Table 3). This is in line with previously published data showing that ~0.6% of the risedronate dose is absorbed when taken after an overnight fast and 4 h before food.^9^ When taken according to the label instructions (30‐min fast before food), the bioavailability of IR risedronate is reduced further. Indeed, Mitchell and colleagues reported that the amount of risedronate secreted in the urine was decreased by a further 55% when taken 30 min versus 4 h before food (indicated by the dashed line in Figure 2).^12^ This effect was also confirmed by another study using 5 mg risedronate in a Japanese population.^22^ Based on this, the A’_e_ of formulation 2 taken in a fasted state (group B) is most similar to IR risedronate when taken according to the dosing instructions.

In the fasted state, formulation 4 disintegrated at the junction of the small and large intestine, whereas formulations 1–3 disintegrated in the small intestine. This was expected, as the luminal pH ranges from 5.5–7.0 in the proximal small bowel, rising progressively to pH 7.5 in the distal regions.^23^ In the fed state, the tablets retained their integrity in the stomach and onset of tablet disintegration was delayed as a result of extended gastric residence. Formulations 1–3 still disintegrated in the small intestine as in the fasted state, whereas the location of disintegration changed from the ascending colon to the small intestine for formulation 4. This is in contrast to IR BPs, which start disintegration in the stomach and are absorbed in the stomach, ileum and duodenum.^24^ This means that a GR formulation allows for targeted release of risedronate in the small intestine, where it may be absorbed more readily than in the stomach.

In the fasted state, the mean A’_e_ of risedronate GR formulation 4 (0.108% [90% CI 0.051–0.231]) was lowest of all GR formulations tested, followed by formulation 3 (0.154% [90% CI 0.090–0.264]) formulation 1 (0.220% [90% CI 0.124–0.389]) and formulation 2 (0.298% [90% CI 0.122–0.730]). The low absorption of formulation 4 may be explained by its disintegration site in the large intestine, where drug absorption is low compared with the small intestine. Formulations 3 and 1 were both coated in 10% methacrylic acid copolymer type C, which may account for their lower mean A’_e_ versus formulation 2 in a fasted state, as there are no significant differences in the disintegration site of these three GR formulations.

The formulations of risedronate tested in this study were well tolerated, with the majority of AEs being mild in severity. The safety profile of all investigated GR formulations was comparable to that previously reported for IR risedronate. There was no substantive trend in distribution of AEs (by severity or causality) among the formulations tested. Musculoskeletal (including myalgia) and GI AEs were the most frequently reported, as would be expected based on the known safety profile of risedronate.

Research on alendronate, another oral nitrogen‐containing BP, indicates that taking this medication with food can reduce its absorption by >85%.^25^ Owing to similarities in their molecular structure, the effect of food on risedronate absorption is likely to be similar. When taken with food, all GR formulations showed a decrease in absorption. This was largest for formulations 3 and 2, which showed an 80% (group F vs. G; 0.20 [90% CI 0.088–0.479]) and 73% (group D vs. E; ratio 0.275 [90% CI 0.082–0.921]) decrease in A’_e_ after food, respectively. The A’_e_ of formulation 1 only decreased by 27% in the fed state when compared with the fasted state (group B vs. C; 0.731 [90% CI 0.282–1.893]). This indicates that this formulation is the one most likely to have efficacy in maintaining bone mineral density when taken with food.

In fact, a clinical trial investigating this formulation of GR risedronate 35 mg showed that it is as effective as IR risedronate 35 mg in maintaining bone mineral density over a 2‐year period in patients with osteoporosis.^26^ This was also found with patients who took the GR formulation with breakfast.^26^ In clinical practice, up to 30% of osteoporosis patients do not fast appropriately before taking their oral BPs.^11^, ^16^ Such non‐compliance may be unintentional, as patients may not remember the dosing instructions they have been given by their physicians.^11^ If such non‐compliance persists, patients may be considered as non‐adherent to their medication, often without the knowledge of their managing physician. Non‐adherence to oral BPs is associated with an increased risk of fractures.^17^ This GR formulation of risedronate has the potential to improve medication adherence by reducing persistent, unintentional non‐compliance and giving patients more flexibility with their dosing schedule.

Indeed, this is supported by findings from a recent retrospective observational study of 5,452 post‐menopausal women from a US claims database: prescription of GR risedronate was associated with a lower incidence of fractures versus other oral bisphosphonates.^21^

Limitations of this study included that the majority of participants were male (75%) and Caucasian (96%). Furthermore, the proportion of men and women differed between groups. Absorption is not expected to differ greatly in women, who are more likely than men to require BP therapy. Non‐Caucasian populations (for example, Japanese) may have some differences in absorption, possibly related to diet,^22^ but these are probably not clinically relevant. Also, this was a parallel group study, so some of the differences seen between treatments may be due to inter‐group variability. The pooled urine sampling method, and the use of a single pharmacokinetic parameter (A’_e_) as the primary variable, does not provide a detailed overview of the pharmacokinetics. In addition, A’_e_ is a surrogate endpoint and represents only a small proportion of the ingested dose.

In conclusion, this study showed that risedronate GR formulation 2 (high 30% methacrylic acid copolymer type C coating, pH trigger 5.5) had the highest absorption when taken fasted, whereas the absorption of risedronate GR formulation 1 (low 10% methacrylic acid copolymer type C coating, pH trigger 5.5) was least affected by the presence of food (−27%).

Patients’ adherence to their osteoporosis medication and compliance with dosing instructions are influenced by many factors, including convenience of the dosing regimen.^21^, ^27^ A GR formulation of risedronate 35 mg that could be taken with food would not only provide a more flexible dosing option to patients, but may also improve adherence and thus has the potential to be more effective in reducing fragility fractures.^4^, ^17^, ^21^, ^28^

## DISCLOSURE

Diane Kleinermans: D.K. does not have any conflicts of interest to disclose. Andrew Joyson: A.J. is an employee of Novartis. Heather Wray: H.W. is an employee of AstraZeneca.

## AUTHOR CONTRIBUTIONS

D.K., A.J., and H.W. have made substantial contributions to conception and design, acquisition of data, analysis and interpretation of data. They have been involved in critically revising the manuscript for important intellectual content. They have given final approval of the version to be published, and agreed to be accountable for all aspects of the work in ensuring that questions related to the accuracy or integrity of any part of the work are appropriately investigated and resolved.

## ETHICS APPROVAL

This study was conducted in line with the ethical principles outlined in the Declaration of Helsinki. Ethics approval was obtained from the Huntingdon Local Research Ethics Committee, Cambridge, UK.

## PRINCIPAL INVESTIGATORS

The authors confirm that the Principal Investigators for this paper are Diane Kleinermans and Heather Wray and that they had direct clinical responsibility for healthy volunteers.

## PATIENT CONSENT

All participants provided written informed consent prior to the study.

## CLINICAL TRIAL REGISTRATION

This trial was conducted before 2013 and was not subject to mandatory registration. In addition, it did not include patients, but healthy volunteers.

## Data Availability

The data that support the findings of this study are available from Theramex Ltd. Restrictions apply to the availability of these data, which were used under license for this study. Data are available from the authors with the permission of Theramex Ltd.
